# Real-world evidence for cladribine tablets in multiple sclerosis: further insights into efficacy and safety

**DOI:** 10.1007/s10354-022-00931-4

**Published:** 2022-04-22

**Authors:** Tobias Moser, Tjalf Ziemssen, Johann Sellner

**Affiliations:** 1 Department of Neurology, Christian Doppler Medical Center, Paracelsus Medical University, Salzburg, Austria; 2 Department of Neurology, Multiple Sclerosis Center, Center of Clinical Neuroscience, Carl Gustav Carus University Hospital, Technical University Dresden, Dresden, Germany; 3 Department of Neurology, Klinikum rechts der Isar, Technische Universität München, Munich, Germany; 4 Department of Neurology, Landesklinikum Mistelbach-Gänserndorf, Liechtensteinstraße 67, 2130 Mistelbach, Austria

**Keywords:** Cladribin, Safety, Efficacy, Real-world data, SARS-CoV‑2

## Abstract

Cladribine (CLAD) is a purine nucleoside analog approved in tablet form to treat highly active multiple sclerosis (MS). CLAD tablets are the first oral therapy with an infrequent dosing schedule, administered in two annual treatment courses, each divided into two treatment cycles comprising 4–5 days of treatment. The efficacy and safety of CLAD tablets have been verified in randomized controlled clinical trials. Clinical observational studies are performed in more representative populations and over more extended periods, and thus provide valuable complementary insights. Here, we summarize the available evidence for CLAD tablets from post-marketing trials, including two observational, four long-term extensions, and two comparative studies. The patients in the post-marketing setting differed from the cohort recruited in the pivotal phase III trials regarding demographics and MS-related disability. The limited number of studies with small cohorts corroborate the disease-modifying capacity of oral CLAD and report on a durable benefit after active treatment periods. Skin-related adverse events were common in the studies focusing on safety aspects. In addition, single cases of CLAD-associated autoimmune events have been reported. Lastly, CLAD tablets appear safe regarding COVID-19 concerns, and patients mount a robust humoral immune response to SARS-CoV‑2 vaccination. We conclude that the current real-world evidence for CLAD tablets as immune reconstitution therapy for treatment of MS is based on a small number of studies and a population distinct from the cohorts randomized in the pivotal phase III trials. Further research should advance the understanding of long-term disease control after active treatment periods and the mitigation of adverse events.

## Introduction

Cladribine (CLAD) tablets were approved for the disease-modifying therapy of highly active relapsing multiple sclerosis (MS) by the European Medicines Agency (EMA) in 2017. Two years later, CLAD tablets were approved by the Federal Drug Administration (FDA) for active forms of MS in adult patients who have had an inadequate response to or cannot tolerate an alternate drug indicated for the treatment of MS. CLAD (2-chloro-2′-deoxyadenosine [2-CdA]) is a purine analog that is taken up into proliferating cells, preferentially into B and T lymphocytes due to their high ratio of activating deoxycytidine kinase to 5′-nucleotidase [1]. The clinical effects are believed to be mediated by a transient reduction of lymphocyte subtypes, followed by a recovery period and return to the normal range [2]. CLAD tablets are taken over a period of 14 months, with two short cycles divided into four courses, and can provide durable efficacy without the potential need for immediate further treatment [3].

In the pivotal trials (CLARITY, ORACLE MS, and ONWARD), CLAD tablets demonstrated significant efficacy versus placebo in reducing relapse rate, disability progression, and magnetic resonance imaging (MRI) worsening in people with MS (pwMS) [4–6]. The subsequent CLARITY Extension study showed long-lasting effects on clinical and MRI outcomes in an additional 96-week follow-up without the need for re-treatment in a subgroup of patients [7, 8]. A sustained, drug-free remission lasting years after CLAD administration is attributed to the pronounced effect on the adaptive immune system [9, 10]. Extensive peripheral immune cell profiling using flow cytometry revealed a selectivity of CLAD-induced depletion towards Th17 cell and memory B cell phenotypes [11, 12]. These subsets play a key role in the pathogenesis of MS [13, 14] and do not recover throughout 24 months, while regulatory subsets expand proportionally [12]. This attempt to selectively deplete immune cell subsets harboring auto-aggressive, encephalitogenic lymphocyte clones is accompanied by modulatory effects of oral CLAD on the overexpression of adhesion molecules and costimulatory receptors on B and Th cells, which are important for immune cell communication and migration [15]. Taken together, there is evidence that oral CLAD impacts dysregulated adaptive immune system functions. Despite temporary but pronounced effects on lymphocyte numbers, systemic, disseminated severe, and opportunistic infections were not reported as significant concerns in the pivotal trials. Only the incidence of herpes zoster infections was increased following treatment with CLAD tablets [16]. The final safety report from the clinical development program, which included additional data from the prospective observational PREMIERE registry, identified no new safety signals among 923 pwMS receiving the approved CLAD dose [17]. Ten cases of malignancies were detected within the follow-up of almost 4000 patient-years, which was numerically higher than but not statistically different from placebo [3]. The rate of observed malignancies with CLAD tablets was not different from the expected rate in the matched GLOBOCAN reference population (standardized incidence ratio 0.88; 95% CI, 0.44–1.69) [17]. CLAD-related skin reactions were rare, and secondary autoimmune events were not reported as a specific side effect of CLAD tablets.

Randomized controlled clinical (RCTs) trials are indispensably associated with the extraordinary progress in MS therapy over the past decades [18, 19]. However, RCTs constitute a well-controlled population under ideal conditions and are not representative of MS cohorts in the real world [20]. Indeed, pwMS considered for clinical trials have to comply with multiple restrictions, including age, disease activity, and pretreatment. Another issue of RCTs is the relatively short observation periods, which fail to provide important information about durable efficacy and long-term safety. Thus, some clinical questions are often better addressed using real-world data reflecting more representative populations and can provide valuable insights into the long-term risks and benefits of therapies [21]. Indeed, less common or delayed adverse events and the cumulative risk of side effects are insights that can be gained by analyzing real-world evidence (RWE).

This review summarizes cohorts treated with CLAD tablets and evaluated for effectiveness and safety under real-world conditions. Moreover, we analyze safety aspects of CLAD therapy, also in the context of the COVID-19 pandemic.

## Real-world evidence for efficacy

Two medium-sized cohort studies comprising a total of 360 patients with MS which evaluated the efficacy of oral CLAD post-approval were identified. Table 1 depicts details of patient demographics and efficacy parameters compared to the cohorts of the pivotal trials.

**Table 1:** Demographics and efficacy outcome parameters from post-approval reports and from the randomized controlled phase III trials (RCT) ORACLE MS and CLARITY. Real-world evidence (RWE) includes two observational studies and four long-term extension trials. Note that data include patients who were switched to other DMTs following CLAD treatment

	Author (year of publication)	Lizak (2021) [24]	Pfeuffer (2021) [22]	Giovannoni (2021) [26]	Yamout (2020) [28]	Moccia (2020) [27]	Patti (2020) [25]	Leist (2014) [4]	Giovannoni (2010) [5]
	Study design	Observ. registry	Observ. prosp.	Long-term extension	Long-term extension	Long-term extension	Long-term extension	RCT	RCT
Demographics	*n* =	90	270	98	22	13	80	206	433
	Female (%)	72	61	68	–	69	58	63	69
	Age (mean ± SD)	47 ± 12	39	38 ± 11	–	39 ± 7	39 ± 10	32 ± 9	38 ± 10
	RRMS (%)	78	100	–	–	–	75	35	100
	BL EDSS (median)	5.25	2.0	3.0	–	3.5	–	1.5	Mean: 2.8
	Median follow-up (months)	42	25	60	118	98	73	24	24
Efficacy	Relapse free at 24 months (%)	65	–	–	–	–	85	–	80
	Relapse free at EOS (%)	–	74	–	–	54	57	–	80
	Free of disability prog. at 24 months (%)	80	–	–	–	–	97	–	86
	Free of disability prog. at EOS (%)	–	76	75	86	39	64	–	86
	Therap. switched at EOS (%)	69	–	–	59	69	68	–	–
	Time to next DMT (median, years)	1.7	–	–	–	–	3.8	–	–
	Time to first relapse (months)	–	9	–	–	–	–	–	13
	ARR after CLAD	0.3	–	–	0.2	0.17	0.19	–	0.14
	ARR before CLAD	1.8	1.0	–	–	–	–	–	–

*Observ.* observational, *prosp.* prospective, *RCT* randomized controlled trial, *n* number of patients included, *SD* standard deviation, *RRMS* relapsing remitting multiple sclerosis, *BL* baseline, *EDSS* Expanded Disability Status Scale, *EOS* end of study, *DMT* disease-modifying therapy, *CLAD* cladribine, *ARR* annualized relapse rate, *prog.* progression, *NEDA* no evidence of disease activity, *IFN* interferon, *GA* glatiramer acetate, *DMF* dimethyl fumarate, *FTY* fingolimod, *NAT* natalizumab, *ALEM* alemtuzumab

A prospective observational study with 270 CLAD tablets-treated patients from two German tertiary care MS centers included the efficacy endpoints of relapse rate, expanded disability status scale (EDSS), and T2 lesions on MRI [22]. The study was carried out between November 2017 and March 2021. The analysis of baseline demographics revealed that the mean age of patients in the real-world cohort was higher, and women were less frequent. Ninety-seven patients (36%) were treatment naïve and 41 were previously on a disease-modifying drug (DMD) for highly active MS (fingolimod [FTY] or natalizumab [NAT]). However, the authors found a substantial reduction in relapse rates after switching to CLAD and of new or enlarging T2 lesions during a follow-up of 36 months compared with pre-CLAD rates. Compared to 279 relapses in the year before the start of CLAD therapy, only 85 occurred after CLAD initiation. The EDSS scores remained stable in most patients during the observation period. CLAD was most efficacious in patients previously without immunotherapy or on therapy with low-potency substances. In contrast, pwMS switched from NAT frequently experienced persistent inflammatory disease activity. In detail, 18/23 of the cohort with prior NAT therapy had clinical or paraclinical disease reactivation, which mainly occurred within 6 months from CLAD commencement. None of the patients previously treated with NAT were switched to CLAD because of unsatisfactory disease control, but for safety reasons, the median washout period was 66 days. Thus, the authors discuss that some cases of continued disease activity could be attributed to a rebound phenomenon in the aftermath of NAT discontinuation [23]. Taken together, this study corroborates a positive impact of CLAD tablets on relapse and MRI activity throughout a mid-term follow-up, except for individuals previously with NAT.

An Australian MS registry study retrospectively analyzed records of 90 pwMS treated with CLAD tablets over a median follow-up of 42 months [24]. Compared with baseline characteristics from the phase III trials, their cohort was older (mean 47 vs. 32 [4] and 38 years [5], respectively) and had a higher Expanded Disability Status Scale (EDSS, median 5.5 vs. 1.5 [4]). Patients had been treated with more than two DMDs before switching to CLAD, and the most commonly used DMDs before CLAD were interferon‑β (IFN‑β, 39%), NAT (20%), and glatiramer acetate (GA, 14%). In addition, 18 patients had secondary progressive MS (SPMS) at initiation of CLAD therapy. Relapse-free survival was present in 65% and no disability progression in 80% by 2 years from initiation of therapy with CLAD tablets. The annualized relapse rate (ARR) of the patients with a relapsing disease course (*n* = 51) decreased from 1.8 to 0.3 after CLAD initiation [24]. Within the observational period of up to 60 months, 69% switched to another DMD after a median of 1.7 years. However, 73% of patients who switched to another DMD did so without the occurrence of relapse. Post-CLAD treatment regimens comprised FTY (29%), dimethyl fumarate (DMF, 19%), NAT (11%), teriflunomide (TERI, 7%), GA (3%), and IFN‑β (2%). In addition, one patient was switched to rituximab, and one treated with autologous hematopoietic stem cell transplantation. Even though the patients only received half of the approved cumulative dose of CLAD in year one, these single-arm prospective data suggest a positive effect of CLAD on disability progression and relapses in patients with advanced disability and age.

## Long-term extension studies

We identified four long-term observational reports of immunotherapy with CLAD tablets; these were follow-ups of participants of the pivotal trials. We here summarize these small to medium-sized studies comprising 213 patients in total.

An Italian registry cohort evaluated 80 patients randomized to CLAD tablets during the pivotal trials [25]. The study retrospectively analyzed time-to first relapse and time-to 12-week sustained disability progression within a follow-up of up to 137 months. At 60 months from last CLAD intake, the probability of relapse-free and progression-free survival was 57 and 64%, respectively. Among the ten patients diagnosed with clinical isolated syndrome (CIS) at the initiation of therapy with CLAD, 60% converted to definite MS. During the follow-up after completing the clinical trial, 68% of the patients started at least one other DMD. The most commonly used agents post-CLAD were IFN‑β, NAT, and FTY. In summary, more than half of the patients did not have any clinical event 60 months from the last CLAD intake, corroborating the durable efficacy.

Encouraging results on a positive impact of CLAD on long-term disability stabilization are deduced from an exploratory post-hoc analysis on 98 CLAD patients receiving the recommended dose of 3.5 mg/kg CLAD during the CLARITY trial [26]. The authors assessed the EDSS course over 60 months as the primary outcome parameter. Five years from randomization for the CLARITY trial, the median EDSS score improved by 0.5 compared with baseline. A worsened EDSS score was found in 24.7%. Of particular note, almost every fourth patient’s EDSS was rated as improved over 5 years from CLAD commencement. Lastly, the authors compared long-term disability progression to a cohort of 186 patients receiving additional treatment cycles in years three and four (cumulative dose of 7 mg/kg). In line with the results from the CLARITY Extension study, higher doses than the approved formulation had no significant impact on long-term efficacy. However, a major caveat of this study is that about 30% dropped out prior to the end of the planned observation period.

A small, single-center retrospective study assessed the risk of disability progression throughout an observational period of 8 years [27]. This study from Italy comprised 13 patients treated with CLAD tablets and 14 enrolled in the placebo arms of phase II and III trials. After reaching the end of the pivotal trial, the patients were treated with different immunotherapies, which were well distributed among the two groups. The patients exposed to oral CLAD at some point during the RCTs performed significantly better in all three primary endpoints consisting of EDSS progression (*p* = 0.017), reaching EDSS of 6 (*p* = 0.036), and conversion to SPMS (*p* = 0.010) compared to the placebo group. In addition, the risk of relapse was significantly reduced for CLAD-treated patients within the first 4 years after CLAD start (*p* = 0.045).

A Lebanese group retrospectively evaluated 22 patients assigned to oral CLAD within the CLARITY trial with a follow-up of up to 10 years [28]. The ARR of 0.20 remained stable during the entire observation period. Only 3 patients (14%) had an increased EDSS compared to baseline and 2 (9%) had converted to SPMS. Even though more than half (13/22) had started a new DMD in the meantime and the final report including MRI features has still to be published, this long-term finding is encouraging for the disability-withholding effect of CLAD.

Taken together, the long-term extension studies provide evidence that oral CLAD has a prolonged impact on disease activity, which appears not to be limited to acute inflammation but also include chronic disability progression.

## Comparison of efficacy with other immunotherapies

We found two comparative trials comprising data from a total of 705 CLAD-treated pwMS. A single-center post-hoc analysis from Canada retrospectively compared the efficacy of oral and intravenous CLAD formulations in 65 patients to 46 on alemtuzumab (ALEM). The patients were followed up for a median of over 3 years and up to 12 years for 420 person-years [29]. At baseline, the CLAD group was older and had more advanced disease (age: 36 years vs. 44 years; baseline EDSS: 3.0 vs. 4.0; years since MS diagnosis: 5 vs. 11). The percentage of individuals retaining a status of no evidence of disease activity (NEDA) at 2 years was higher with ALEM (80.4% vs. 65%, *p* = 0.023). Moreover, relapses occurred significantly earlier after CLAD therapy (median 6 months vs. 32 months; *p* = 0.0025). Beyond 2 years after treatment start, however, no statistically significant differences were found regarding the likelihood of event-free survival. A new immunomodulatory therapy was started due to disease activity after year 2 in 19.6% and 18.5% for ALEM and CLAD, respectively.

Another comparative study was carried out by an Italian group, merging data from the CLARITY trial with real-world data from newly diagnosed patients (Italian multicenter database, i‑MuST) [30]. The key endpoint of this observational retrospective study was the ARR over 2 years in patients treated with CLAD (*n* = 640) compared to patients on low- to moderate-efficacy DMDs including IFN‑β (*n* = 1168), GA (*n* = 402), and DMF (*n* = 295), and on escalation therapies including FTY (*n* = 113) and NAT (*n* = 149). Importantly, the baseline characteristics were well balanced between the different treatment groups. CLAD therapy was associated with significant reductions in the ARR compared to DMDs used for the treatment of mild to moderate disease activity (52% vs. IFN‑β [*p* < 0.001], 51% vs. GA [*p* < 0.001], and 40% vs. DMF [*p* = 0.001]). The relapse ratio (RR) was similar to that of FTY patients (RR = 0.74; *p* = 0.24) and inferior compared to NAT (RR = 2.13; *p* = 0.014). Of note, the effect of CLAD on relapses was even better in the subgroup of patients experiencing higher disease activity at baseline.

## Real-world evidence for safety

Insights to post-approval safety for CLAD tablets were provided by studies from two German MS centers [22, 31]. These studies prospectively evaluated more than 200 patients with a median follow-up of 2 years. The studies disclosed a high overall incidence of CLAD-associated skin reactions [31]. Every third patient (32%) was affected by at least one dermatological adverse event (AE). Skin reactions most commonly occurred within 3 months from CLAD administration (70%), and the predominant manifestations were hair thinning (11.7%) and skin rash (8.4%). Other frequent acute skin reactions included pruritus (2.6%) and transient mucositis (5.4%). Of the latter, 2 patients developed a dental abscess. Acute skin AE were generally temporary and resolved either spontaneously or following treatment with steroids and/or antihistamines. The exceptions were 9 patients who had manifestations that persisted throughout the study period (*n* = 6 hair thinning, *n* = 3 rash). Apart from this study cohort, an acute drug hypersensitivity has been reported in a male patient who developed lichenoid eruptions in close relationship to each CLAD cycle [32]. After treatment with steroids and antihistamines, the acute skin rash resolved within 40 days.

Delayed skin AEs, defined as occurrence more than 3 months from of any CLAD intake, were less frequent (2.9%). They included, besides rash and hair thinning, alopecia areata in two individuals (27- and 34-year-old women) and leukocytoclastic vasculitis (42-year-old woman) in one case [31]. Alopecia was diagnosed at 13 and 20 months from CLAD start and persisted throughout the follow-up. The clinical manifestation of vasculitis was at 22 months from the first CLAD intake and improved with oral steroids. Since no other explanatory triggers were identified, the authors hypothesized that these AE are autoimmune complications related to CLAD therapy [31]. Another possible secondary autoimmune AE following treatment with CLAD is a case of histologically proven antibody-mediated glomerulonephritis (44-year-old woman) [33]. Shortly after the fourth CLAD cycle, she developed renal failure and subsequently hemolysis and thrombocytopenia. Despite intensified treatment with pulsed steroids, plasmapheresis, and eculizumab, this patient remained hemodialysis dependent. Taken together, isolated cases of autoimmune events following CLAD have been reported since drug approval, but a causative relationship remains to be confirmed.

The evaluation of the safety data generated in the pivotal trials revealed that the AE of special interest was most frequently the occurrence of herpes zoster, followed by oral herpes and herpes simplex [16]. In a bicentric study from Germany, herpes virus infections occurred in 14.6% at a median of 83 days (range 10–305 days) after the intake of CLAD tablets [22]. In this cohort, the herpes virus infections related to both herpes-simplex virus (HSV) and VZV, and mostly occurred in year 1. Two patients had cranial nerve involvement among the 22 patients with VZV infection. No VZV encephalitis case was present in the cohort. While most HSV infections resolved following local treatment, all patients with VZV infection received intravenous acyclovir. Interestingly, the incidence of VZV reactivation but not that of HSV infection was significantly associated with low lymphocyte counts. Indeed, lymphocyte numbers were decreased among all patients during VZV manifestation, and every second patient had lymphopenia grades 3 and 4 (< 500 cells/µl) [22]. In line with this, lymphocyte counts at manifestation of HSV disease (median 860 cells/μL; range: 420–1150 cells/μL) were lower for VZV infection (median 570 cells/µl; range: 220–1120 cells/μL) [31]. Of note, previously DMF-exposed patients were more prone to develop severe lymphopenia (odds ratio: 5.0; *p* < 0.001) compared to the remainder and had mainly VZV infections [22]. Interestingly, the grade of lymphopenia in DMF-pretreated individuals was independent of the baseline lymphocyte count. Even though the data are insufficient to give recommendations on whether and when to consider antiviral prophylaxis, the authors conclude that patients switching from DMF to CLAD should be closely monitored for and eventually (booster) vaccinated against VZV. Interestingly, the incidence of VZV infection was much lower in an observational study from Canada (2%), which retrospectively compared the safety profile of ALEM (*n* = 46) and CLAD (*n* = 65; tablets and infusions) over 3 years [29]. The CLAD-treated group was older (*p* = 0.0002), with higher baseline EDSS (*p* = 0.0015), and more likely to be secondary progressive (*p* < 0.0001). They found better tolerability of CLAD, and ALEM was associated with significantly more infusion-related AE (80% vs. 17% for the IV CLAD treatment), VZV infections (22% vs. 2%), and secondary autoimmune events (56% vs. 3%). Interestingly, the authors classified two cases of hypothyroidism (3%) as autoimmune adverse reactions linked to CLAD treatment [29]. This study also addressed the occurrence of malignancies. These were equally distributed among the two cohorts (2% each) and included follicular lymphoma (ALEM) and recurrent basal cell carcinoma (CLAD). In contrast, the integrated safety analysis of the CLAD development program recorded ten malignancies in 3754 patient-years compared to only three events among the placebo cohorts (2275 patient-years) [3]. CLAD-associated malignancies consisted of melanoma (*n* = 2) and one case each of basal cell carcinoma, squamous cell carcinoma, pancreatic, breast, ovarian, rectal, and papillary thyroid cancers, and bile duct adenocarcinoma. The wide spectrum indicates no clustering of any particular cancer type. Importantly, the incidence rate of malignancies under CLAD tablets was not different from the patient cohort treated with other DMDs [34]. Although signals of carcinogenicity were ultimately disproved, long-term monitoring has been recommended by the EMA and the FDA [35]. Due to the relatively short follow-up period in the pivotal trials compared to a long latency period for many cancers [36], real-world data over many years are warranted to detect potential cancer side effects associated with immunotherapies. The German study (*n* = 239) detected cancerous skin lesions (squamous cell carcinoma, *n* = 2) in 0.8% of pwMS treated with CLAD tablets [31]. For now, there is no evidence underscoring an increased rate of neoplasms following CLAD tablet therapy compared to the general population. The final safety analysis from the clinical development program, including the PREMIERE registry, confirmed the low level of serious treatment-emergent AEs associated with CLAD tablets [17].

## Real-world evidence for CLAD tablets and COVID-19

There is apparent interest in understanding the risk of an unfavorable COVID-19 outcome and efficacy of vaccination against SARS-CoV‑2 in CLAD-treated patients [37]. Humoral responses to SARS-CoV‑2 vaccines were unimpaired in CLAD-treated patients [38], and postvaccination seropositivity was independent of lymphocyte counts and age [39]. Importantly, antibody titers remained sustained for 6 months postimmunization [40]. Similarly, CLAD appears not to affect pretreatment antibody levels to common pathogens [41]. Moreover, CLAD treatment was not associated with worse or fatal COVID-19 courses [42, 43]. Therefore, existing literature findings suggest no negative impact of CLAD on vaccine responses and COVID-19 outcomes.

## Conclusion

The available early RWE corroborates the efficacy of oral CLAD in a comparable range to the observations from the pivotal trials. While these data are derived from small cohorts and there are restrictions concerning the study design, there is evidence for beneficial effects on relapses and disability progression for up to 10 years. Similarly, the explanatory power of head-to-head studies is limited, and the findings need to be confirmed in larger cohorts with appropriate study designs. At the same time, CLAD generally proved safe under real-world conditions, with a non-negligible incidence of skin reactions and herpes virus infections. Nevertheless, caution is advised for NAT and for DMF switchers regarding efficacy and safety, respectively. Lastly, anecdotal cases have associated secondary autoimmune events with CLAD tablets.

To conclude, we provide first insights into the real-world experience of oral CLAD in MS, derived from patient cohorts with more diversified baseline characteristics and followed over a longer time period. Further studies will be necessary to evaluate the efficacy of CLAD patients after active treatment periods and elucidate the long-term safety of this emerging treatment option.
